# Methods for the Detection of Seizure Bursts in Epilepsy

**DOI:** 10.3389/fneur.2019.00156

**Published:** 2019-02-27

**Authors:** Udaya Seneviratne, Philippa Karoly, Dean R. Freestone, Mark J. Cook, Ray C. Boston

**Affiliations:** ^1^Department of Medicine, St. Vincent's Hospital, University of Melbourne, Melbourne, VIC, Australia; ^2^Department of Neuroscience, Monash Medical Centre, Melbourne, VIC, Australia; ^3^Department of Medicine, School of Clinical Sciences at Monash Health, Monash University, Melbourne, VIC, Australia; ^4^Department of Biomedical Engineering, University of Melbourne, Melbourne, VIC, Australia

**Keywords:** EEG, bursts, seizure cluster, prediction, epilepsy, interseizure interval

## Abstract

**Background:** Seizure clusters and “bursts” are of clinical importance. Clusters are reported to be a marker of antiepileptic drug resistance. Additionally, seizure clustering has been found to be associated with increased morbidity and mortality. However, there are no statistical methods described in the literature to delineate bursting phenomenon in epileptic seizures.

**Methods:** We present three automatic burst detection methods referred to as precision constrained grouping (PCG), burst duration constrained grouping (BCG), and interseizure interval constrained grouping (ICG). Concordance correlation coefficients were used to confirm the pairwise agreement between common bursts isolated using these three automatic burst detection procedures. Additionally, three graphical methods were employed to demonstrate seizure bursts: modified scatter plots, staircase plots, and dropline plots. Burst detection procedures are demonstrated on data from continuous intracranial ambulatory EEG monitoring in a patient diagnosed with drug-refractory focal epilepsy.

**Results:** We analyzed 1,569 seizures, from our assigned index patient, captured on ambulatory intracranial EEG monitoring. A total of 31, 32, and 32 seizure bursts were detected by the three quantitative methods (BCG, ICG, and PCG), respectively. The concordance correlation coefficient was ≥0.99 signifying considerably stronger than chance burst detector agreements with one another.

**Conclusions:** Bursting is a quantifiable temporal phenomenon in epilepsy and seizure bursts can be reliably detected using our methodology.

## Introduction

Epileptic seizures tend to congregate at certain time points of the temporal course in some patients and this phenomenon is broadly defined as seizure clustering (1–3). Identifying patients with seizure clusters is of practical importance as it appears to be a marker of intractable epilepsy (4). However, there is no uniform agreement among investigators, either in the definition or in the quantification of seizure clustering. Furthermore, clustering generally refers to clinical seizures and it has been well-demonstrated that patients underreport their seizures while there may be many subclinical seizures only detected on the EEG (5). Hence, the temporal congregation of seizure activity is much broader than clinical seizure clustering and we have identified this phenomenon as seizure bursts (6).

Patterns of human epileptic seizures and neuronal spike trains share similarities such as specific durations of events (seizures or spikes) and inter-event intervals. Therefore, useful insights can be gleaned from studies on neuronal spikes. For many years, and for good reasons, “burst event complexes” have preoccupied investigations into neuronal spike trains (7). The main motivations for this have been the desire to translate visible features of spike trains into distinct subpopulations and to explore whether clinical or biochemical forces play a role in spike patterns (7). Turnbull et al. (7) offer compelling justifications for the investigation of bursts ranging from their significance in signal encoding to their potential importance as a regulatory mechanism. For neuronal bursting, it has been suggested that inhibitory safeguards may impose an overwhelming accumulation of corrections or adjustments, possibly in association with regional neural structures (7). In conjunction with this, an array of ingenious burst detection methods for neuronal spike trains has been proposed by several researchers (7–10). However, these methods have not yet been translated for the analysis of epileptic seizures and discharges.

At the heart of most burst detection methods is the notion that “if bursts exist amongst neuronal spike trains, then their presence will be highlighted by abrupt changes in spike rate patterns (e.g., shortening of the interspike intervals) distinguishing them from the background” (10). The challenge is to find a way of locating the points (onsets) and the extents (durations) of these abrupt changes. Some key quantitative features of bursts need to be specified before using automated detection methods and there is no consensus on values for these features. Such features have been delineated in previous research and typically include the number of firings per burst and the maximum interspike interval (7, 10, 11).

Similar to clustering, the bursting of epileptic seizures may have clinical implications. Therefore, further characterization and analysis of seizure bursts in humans is warranted. We have previously presented a demonstration of seizure bursts in a cohort of drug-resistant focal epilepsy (6). However, this previous work did not detail the statistical methodology of different techniques for the selection of bursts as being distinct from other seizures and epileptic activity. To ensure further accounts of seizure bursts are robust, it is necessary to present a suite of statistical measures to quantify and detect bursting which remains the focus of the current study. Using intracranial ambulatory EEG data in drug-resistant focal epilepsy, we present qualitative and quantitative methods to identify, characterize, and quantify seizure bursts.

## Materials and Methods

### Subjects and Data

We have previously presented an analysis of the bursting phenomenon in a dataset of continuously recorded intracranial EEG from 15 subjects (6). The aim of the current work is to provide a detailed statistical account and comparative analysis of burst detection methods. The following sections outline the burst detection algorithms. For the purposes of demonstration, burst detection methodology is discussed in detail with reference to a single subject from the previous analysis. Additionally, in order to display the wider applicability of our methodology, we will present the burst-detection graphs of all subjects with demonstrable seizure bursts in the cohort. In view of space limitations though, tables will only be presented in regard to data from our index case.

We used data from a clinical trial of an implantable seizure advisory device as detailed in a previous publication (5). In brief, all subjects in the study suffered from drug-resistant focal epilepsy. Two intracranial electrode arrays with a total of 16 platinum-iridium contacts were implanted over the epileptogenic zone enabling long-term ambulatory EEG data acquisition with wireless transmission to an external device. The EEG seizure detection was automated using an algorithm based on an unsupervised learning approach and all seizures were subsequently verified by expert investigators in conjunction with seizure diaries and audio recordings of the device. The reader is referred to Cook et al. (5) for more details. The current analysis used seizure-onset times marked by this previous study. We wish to emphasize that our burst detection methodology is not designed for automated seizure detection. In order to apply our technique of burst detection, seizure onsets have to be pre-marked by another method, either automated or manual.

Seizures were classified into three groups based on clinical symptoms and EEG characteristics: type 1, 2, and 3. Type 1 events were characterized by clinical symptoms, verified by the seizure diary, accompanied by an electrographic ictal rhythm. In type 2 events, the EEG ictal rhythms were identical to type 1, but without any verified clinical symptoms. Type 3 events were not accompanied by clinical symptoms and the EEG demonstrated an ictal rhythm dissimilar to types 1 and 2.

We selected the subject with the highest mean monthly seizure rate from the study cohort to demonstrate our methods of burst detection. We hypothesized that such a patient is more likely to have seizure bursts given the known association of seizure clusters and refractory epilepsy. We analyzed all clinical (type 1), clinically equivalent (type 2), and subclinical (type 3) seizures captured on the continuous intracranial ambulatory EEG monitoring.

This study was approved by the Human Research Ethics Committee of St. Vincent's Hospital, Melbourne, Australia. Written informed consent was obtained from all the participants of this study. All data were analyzed using Stata software version 14.2 (StataCorp, Texas, USA).

### Burst Detection Methods

In the absence of previous work on seizure bursts, our methodology was heavily influenced by the research on neuronal spike bursts. The burst detection in neuronal spike trains described in previous research belongs to four classes, quantitative methods for the detection of sudden changes in the spike rate (7, 8, 10–13), graphical methods (7, 8, 12, 14), non-parametric methods (9), and methods developed around electrophysiology and inter-neuronal communication as opposed to empirical methods (15, 16).

We adopted three graphical methods to demonstrate the presence of bursts in seizure patterns and three quantitative methods to detect, characterize, and quantify seizure bursts. Additionally, we adopted two approaches to assist with confirmation of the validity of our burst detection methods: one to illustrate the agreement between our bursts with graphical evidence, and one to demonstrate the concordance of pairs of our burst predictors.

We characterized bursts in terms of the number of seizures per burst, the type of seizures (clinical, clinically equivalent, or subclinical) within the bursts, the interseizure interval (ISI), and the duration of the bursts *per se*. Events that constituted a burst (e.g., seizure) were termed “burst elements”. We examined the impact of detection method on statistical summaries of these features. Technical and operational details of our burst detection methods are summarized in Appendix 1.

#### Graphical Methods

Three plot styles were developed to help with our analysis of burst detection by exposing their presence in plots.

##### Modified scatter plot

The modified scatter plot allows us to see the degree to which burst elements align along study time (x-axis) in terms of seizure durations and ISIs (two distinct y-axes). If our speculation about bursts being comprised of homogeneous elements occurring in close proximity to one another, we should see (near) vertical lines with each line representing a burst. We have labeled those vertical assemblies of observations (seizures/ISI's) as “Palisades”.

##### Staircase plot

Our second display is the “staircase plot” where we plot seizure onset time vertically (y-axis) vs. the sequence of seizure occurrence (x-axis) and if bursts exist they will be represented by horizontal plot lines. Conversely, vertical lines with few points (seizures) will coincide with relatively quiescent periods during the study. When bursts and non-burst periods alternate in a subject during the total period of EEG recording, we expect to see a “Staircase pattern” created by alternating vertical and horizontal lines.

##### Dropline plot

Thirdly, we present the plot of seizure duration (y-axis) vs. time into the study when the seizure took place (x-axis). Note that the duration of each seizure is portrayed by the height of the vertical lines. In keeping with the method described by Turnbull et al. (7), we should see dense areas across the plot (“Density bands”) where bursts exist due to the congregation of short, close seizures of similar duration around certain time points.

#### Quantitative Methods

Three automatic burst detection methods were developed and were referred to as precision constrained grouping (PCG), burst duration constrained grouping (BCG), and interseizure interval constrained grouping (ICG).

In all methods, 10 seizures were defined as the lower limit of events per burst. There are two major reasons for our selection of 10 seizures as the smallest permitted composition of bursts. First, previous researchers have indicated numbers of event counts near to this value as the lower limits of spike bursts (7, 10, 11, 13). Second, for statistical analysis of the composition of bursts, 10 events would be ideally placed as the entry level for characterization of the burst “ingredients”.

##### Precision constrained grouping method

This automated grouping method deploys a precision controlled transformation of seizure onset times to achieve their grouping. By this, we mean that when seizure onsets are sufficiently close in time, the transformation of the times may lead to groups of them being judged to appear, computationally, as occurring at the same actual points in time thus rendering bursts.

##### Burst duration constrained grouping method

The second approach to automating burst detection used integer “floored” division to produce limited value (by flooring the calculation) quotients. In this process, the numerator and denominator of the quotient were, respectively, the seizure onset time and the maximum burst duration. The quotients then served as the basis for seizure grouping.

##### Interseizure interval constrained grouping method

Turnbull et al. (7) proposed a dual constraint method for burst detection in the investigation of spike trains. Constraint 1: a minimum number of spikes required for a sequence of spikes to comprise a burst, and constraint 2: a maximum interspike interval to allow blockage of “remote” spikes from being admitted into a burst, at either its beginning or its end. Our implementation of this approach for seizures follows Turnbull et al.'s suggestion with constraint values adjusted to match the seizure setting, as opposed to spikes.

#### Methods to Examine the Agreement Amongst Burst Detectors

We describe two methods to address the agreement among (1) quantitative isolation of bursts and the Palisades in the scatter plots, and (2) the pairwise concordance of the burst onset predictions amongst the three quantitative procedures.

##### Burst-Palisade plot

If the Palisades in our modified scatter plots actually coincide with our predicted burst onsets from the quantitative methods we should be able to conceive a plot showing the two structures overlapping.

We have termed this graphical approach the Burst-palisade plot and here we show seizure duration in seconds (y-axis) against time into the study in days (x-axis). Because we have already shown that the ISI and durations align there is no advantage of plotting ISI, hence the selection of only seizure duration for our plot. In the burst palisade plot, we portray our quantitatively determined burst onsets as thin vertical lines emanating from the x-axis at each predicted burst.

##### Concordance correlation coefficient

Concordance correlation coefficient allows us to confirm the pairwise agreement between common bursts isolated using our three automatic burst detection procedures. For example, comparing PCG and BCG burst onset predictions then, using the approach of Lin (17), if both methods predict the same bursts, we would anticipate their concordance correlation coefficient to be relatively high (i.e., close to “1”) (17).

## Results

### Subject Characteristics

A total of 15 subjects underwent intracranial EEG monitoring in the original study (5). Six subjects displayed seizure bursts when tested with our methods. To detail the methodology, we selected one patient (identification number 3) with drug-resistant focal epilepsy who underwent ambulatory intracranial EEG monitoring for 523 days and a total of 1,569 seizures were captured (type 1 = 160, type 2 = 137, type 3 = 1,272).

### Evidence of Bursts Using Graphical Methods

**Figure 1:**
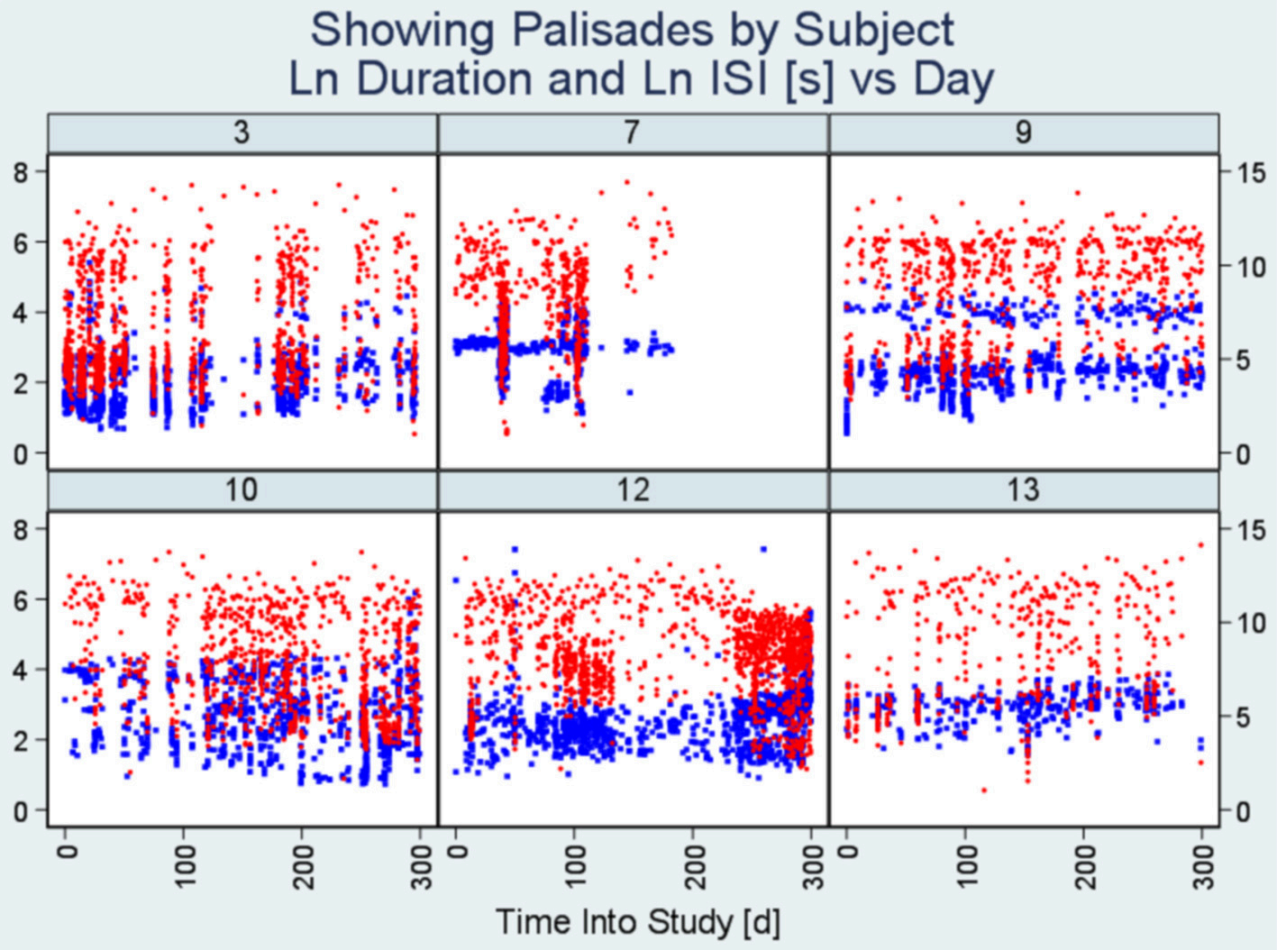
Modified scatter plots. Note there are vertical assemblies of data points constituting seizure durations (blue points) and interseizure intervals (red points). These vertical assemblies referred to as “Palisades” represent the bursting phenomenon. Each plot represents data from an individual patient and the number on top is the unique identification number of the subject. The x-axis represents time into study (in days) whereas the y-axis on the left indicates seizure duration (seconds) and the y-axis on the right represents interseizure interval (seconds). (ISI, interseizure interval; Ln, logarithmic normal).

**Figure 2:**
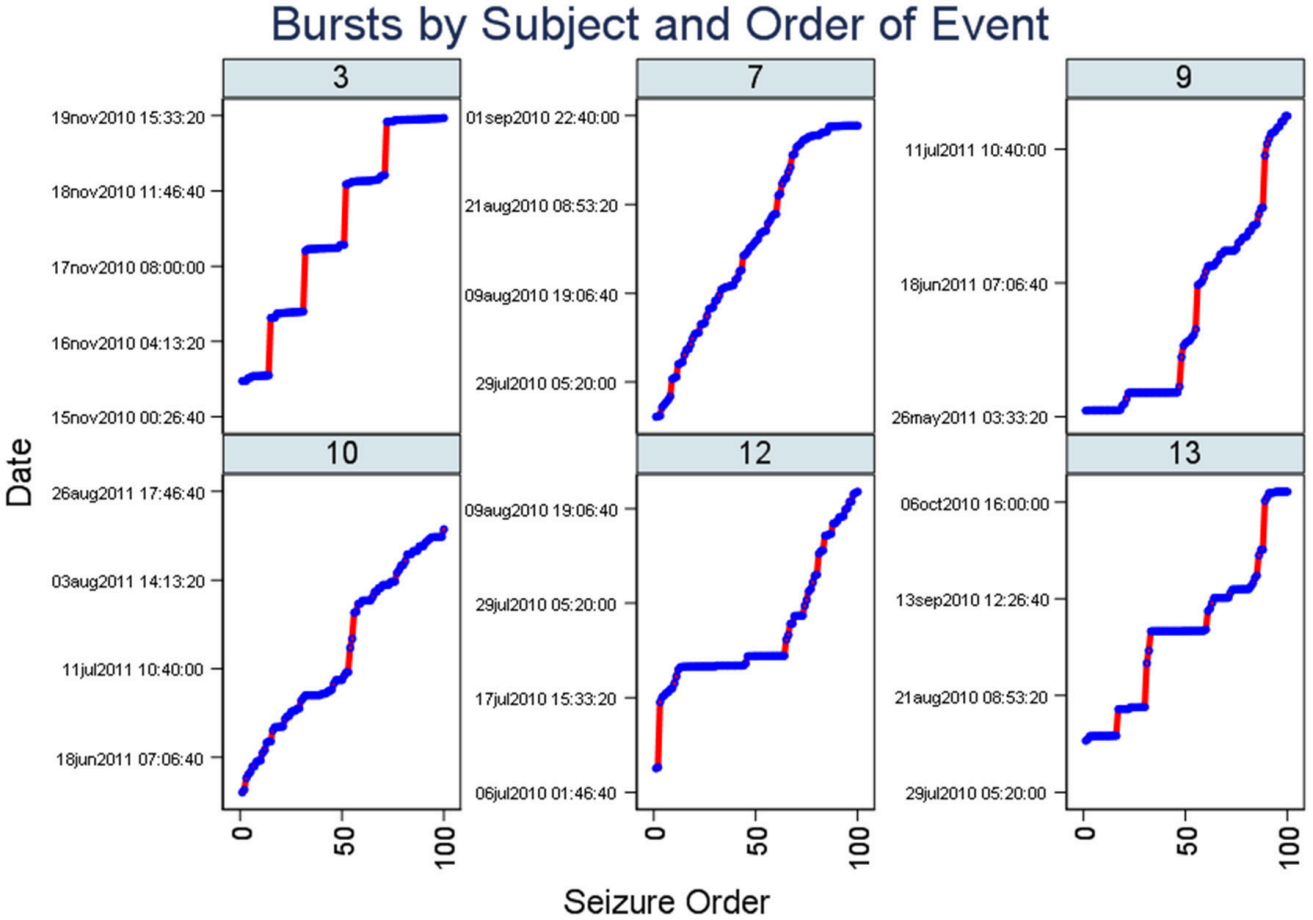
Staircase plots. Note there are segments of dotted horizontal lines and around five vertical lines (“staircase pattern”). The horizontal lines coincide with bursts. Similar to Figure 1, plots from six subjects are included here. The x-axis represents the occurrence of seizures in the chronological sequence whereas the y-axis indicates the date of the study.

**Figure 3:**
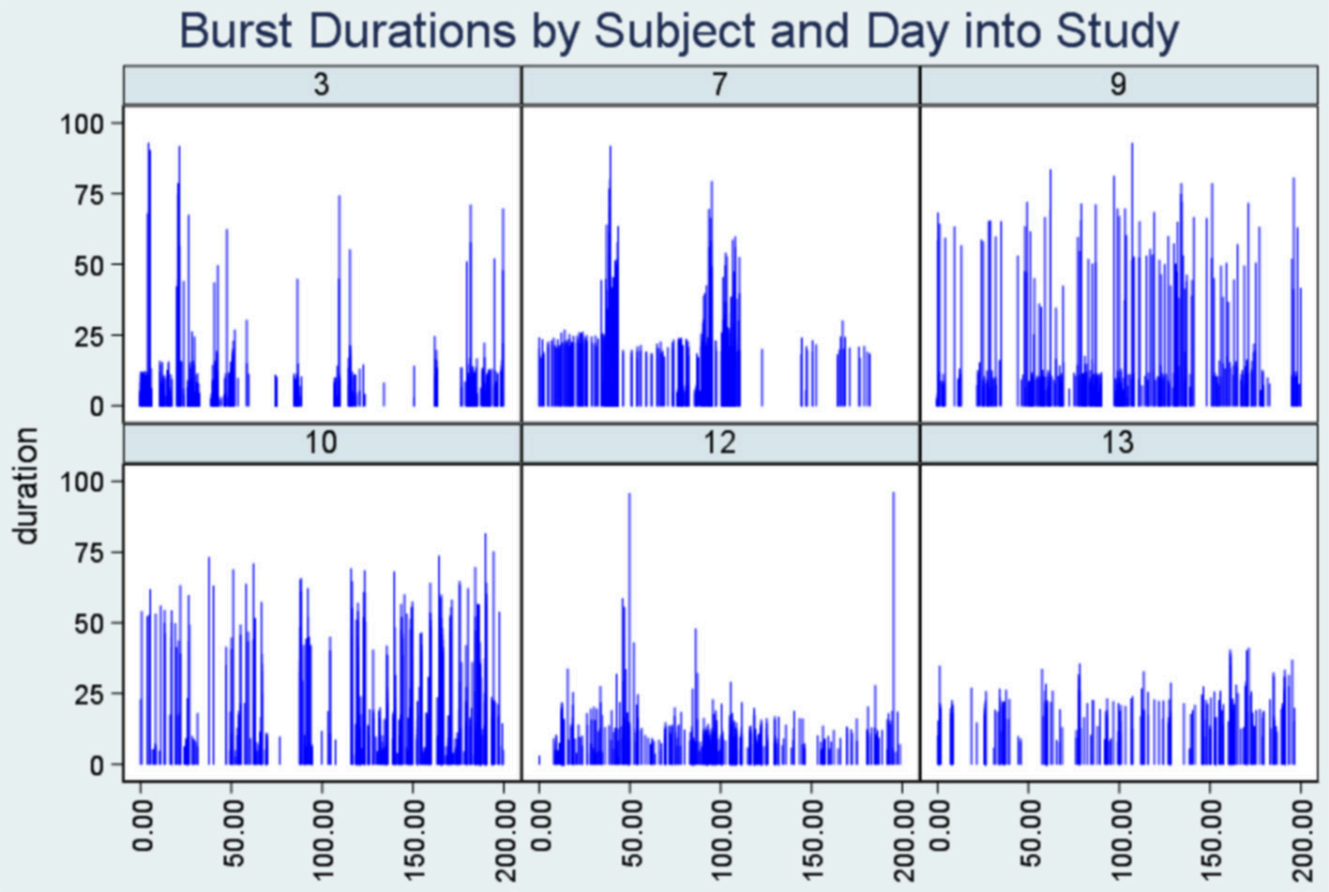
Dropline plots. Note the highly dense horizontal region (“density band”) representing seizure bursts in six patients. The y-axis indicates seizure duration and the x-axis represents time into the study when the seizure took place.

Figures 1–**3** present plot segments using each of the three burst exposure plot styles. In Figure 1 (modified scatter plot) for ID 3, we see that there are around 20 heavy vertical point assemblies comprising seizure durations (represented by blue dots) and ISIs (represented by red dots) coinciding with data palisades (“Palisade pattern”) representing seizure bursts for this subject. In the same figure, we also present the modified scatter plots of the other five patients demonstrating the palisade pattern indicative of seizure bursts.

Figure 2 (Staircase plot) displays, for the selected patient (ID 3), around five segments of dotted horizontal lines and around five vertical lines (“Staircase pattern”). The horizontal lines coincide with bursts. The vertical lines coincide with quiescent periods with few seizures and long ISIs. Staircase plots from the other five subjects are additionally included in this figure.

Lastly, Figure 3 (Dropline plot) illustrates consistency with the conjecture of Turnbull et al. (7) that plot density in such a graphic is evidence of bursting. The low lying, high dense horizontal region (“Density band”) represents seizure bursts, and it is particularly evident in the plots for subjects seven and nine.

We show that, in combination, the burst evidence plots complement one another as shown by the close delineation of a number of seizure bursts in the study (Figure 1) and proximity of bursts (Figure 2). Features from these plots help with burst onset isolation using our quantitative methods as detailed in the next section.

### Evidence of Bursts Using Quantitative Methods

We present data from the patient ID 3 to expose key characteristics of bursts. Table 1 highlights two aspects of bursts; (1) their typical composition in regard to seizure count, and (2) the diversity of seizure types within bursts. The implication of the burst detection method in regard to these aspects is also shown.

**Table 1:** The mean, maximum, and standard deviation of the number of seizures per burst, and the total number of seizures (*n*) in all bursts, broken down by detection method, and by type of seizures.

**Method**	**Seizure type**	**Mean**	**Maximum**	**Standard deviation**	**Total number**
BCG	1	20.0	20.0	0.0	5
	2	20.3	24.0	4.8	28
	3	14.8	24.0	4.0	405
ICG	1	19.0	19.0	0.0	4
	2	20.8	23.0	2.2	27
	3	16.4	26.0	4.9	473
PCG	1	11.0	11.0	0.0	3
	2	15.9	19.0	4.0	18
	3	15.3	24.0	3.9	441

*BCG, burst duration constrained grouping; ICG, interseizure interval constrained grouping; PCG, precision constrained grouping*.

Of particular note is that there are relatively few type 1 and 2 seizures amongst the burst elements (<1 and <5%, respectively) and that the mean seizure count number in regard to type 3 seizures is closely reflected by each detection method. We also infer that for each detection method, each type 1 seizure occurred in the same burst (mean seizure precisely integral, and sd = 0), for example, all 5 type 1 seizures detected using the BCG procedure occurred in a burst containing 20 seizures and further exploration revealed that all such bursts by method were precisely (temporally) adjacent to one another.

Table 2 shows statistics relating to the features (duration and ISI in seconds) of seizures within the burst (burst elements) and it is evident that there is good agreement amongst the different detectors. It would be representative to state that the typical burst element (seizure within a burst) has a duration of 6 s and an ISI of 110 s with durations up to almost a minute and ISIs of 14 min. The constraint of the ICG detection method (ISI < 600 s) precluded from reporting the same maximum ISI as the other methods.

**Table 2:** Mean, median, standard error, minimum, maximum, and, count (*N*) of all burst-related seizures, for seizure duration (upper 3 rows) and for interseizure interval (lower 3 rows), for each detection method.

**Method**	**Mean**	**Median**	**SE of mean**	**Minimum**	**Maximum**	***N***
BCG	6.32	5.09	0.18	2.44	55.23	438
ICG	6.43	5.19	0.14	2.44	21.28	504
PCG	5.99	4.85	0.16	2.44	55.23	462
BCG	108.12	74.26	5.45	4.31	843.12	407
ICG	111.78	76.34	4.55	6.01	564.97^*^	471
PCG	109.29	75.03	5.16	4.31	812.43	430

BCG, burst duration constrained grouping; ICG, interseizure interval constrained grouping; PCG, precision constrained grouping; SE, standard error. Seizure feature time units are seconds. An asterisk (^*^) denotes an active detector, ICG, constraint with the consequence of a maximum interseizure interval (ISI) limitation (<600 seconds). Note that for each burst, there is one fewer ISI than duration value, for example, whereas, for the BCG predictor there are 438 total seizure duration values there are only 407 (=438-31) ISI values.

Table 3 captures the statistics of burst duration (in seconds). A good agreement between the BCG and PCG detectors can be seen, but the ICG detector has a wider range of values. Typically, a burst lasts for about 25 min and the duration ranges from about 12 min to 40 min. Whereas, the BCG detector constraint became active here, this seems to have actually brought the BCG and PCG detectors closer together.

**Table 3:** Mean, median, standard error, minimum, and maximum of the burst duration for each detection method.

**Method**	**Mean**	**Median**	**SE of mean**	**Minimum**	**Maximum**
BCG	1521.36	1531.54	21.94	720.62	2351.49^*^
ICG	1767.29	1420.79	32.25	785.28	3339.06
PCG	1584.29	1495.18	23.48	314.01	2438.08

BCG, burst duration constrained grouping; ICG, interseizure interval constrained grouping; PCG, precision constrained grouping; SE, standard error. Burst duration units are in seconds. An asterisk (^*^) denotes an active detector, BCG, constraint consequence (<2,400 s).

### Evidence of Agreement Among Different Burst Detection Methods

Using our operational methods for burst detection, as detailed in Appendix 1, in subject ID 3, we found that for the detectors BCG, ICG, and PCG, the numbers of bursts detected were, respectively 31, 32, and 32 (an agreement of better than 95%). However, there is no assurance that the methods should each find specific bursts in precisely the same order. Indeed, to achieve this was not included amongst our detection design criteria. To address this in regard to corroboration of bursts across the methods, we developed a “soft sentinel seeking” approach where for each burst located using any one of the detection methods, we scanned the entire array of bursts found using the other methods for a “match” (to <1% tolerance) to the “reference” burst (i.e., sentinel). The scanning match element (or burst sentinel) was based on the mean of the actual seizure onset times in each burst, thus offering the most rigorous basis for the agreement being dependent on all seizures within each burst. The sentinel units were expressed as day (i.e., to the precision of a fraction of a day) of the seizure into the study following the day after the first seizure during the study.

#### Burst-Palisade Plots

**Figure 4:**
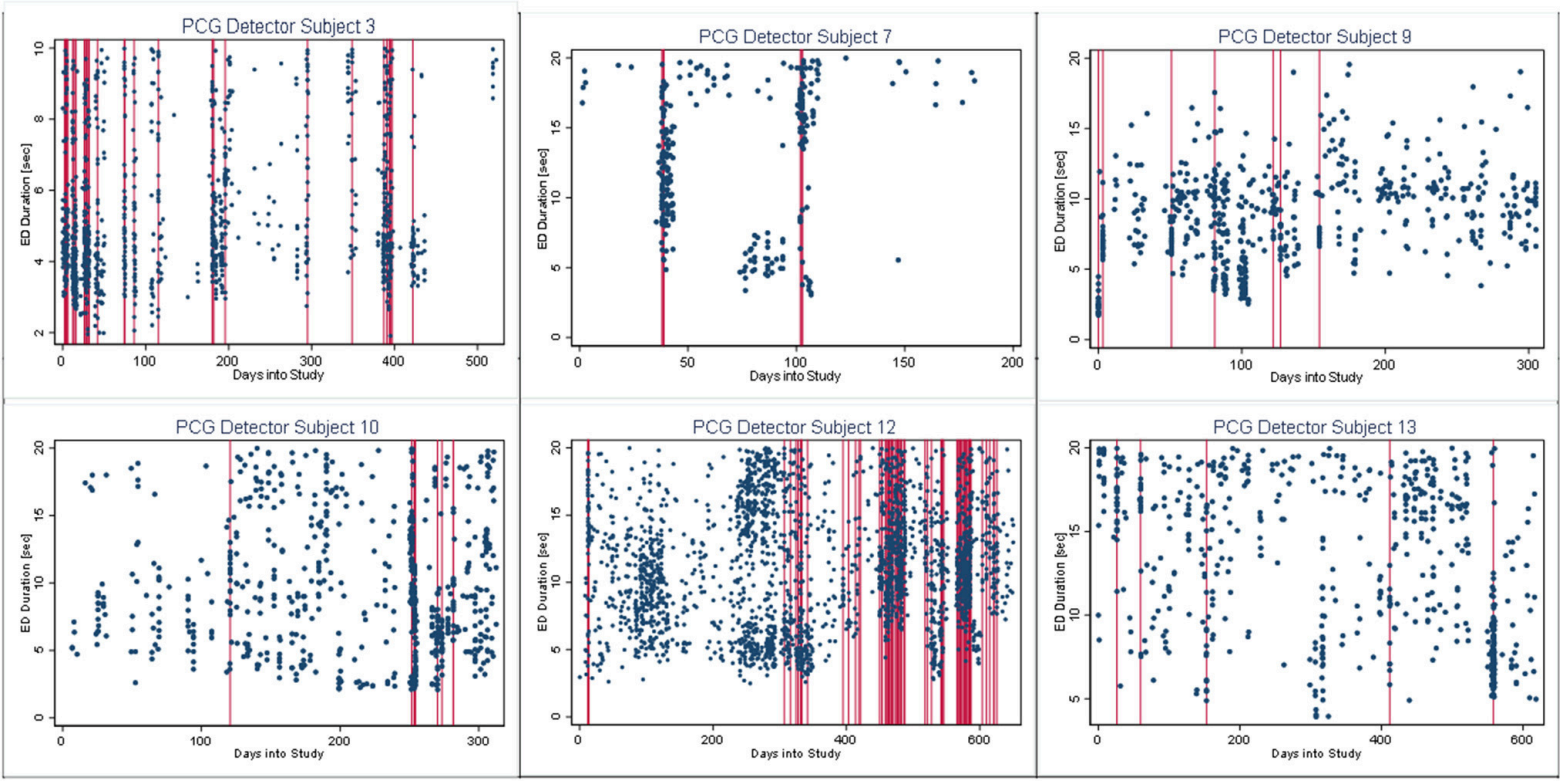
Burst-palisade plot of precision constrained grouping detector. The dots represent the observed seizures against time into the study. The vertical lines in red color represent the predicted burst onsets from burst detection algorithms (PCG). The agreement of the burst prediction algorithms with the modified scatter plot is reflected by the alignment of the vertical lines and the palisades of dots. Data from six subjects who demonstrated seizure bursts are plotted here. Time into the study (days) is on the x-axis, whereas the y-axis represents seizure duration (seconds).

**Figure 5:**
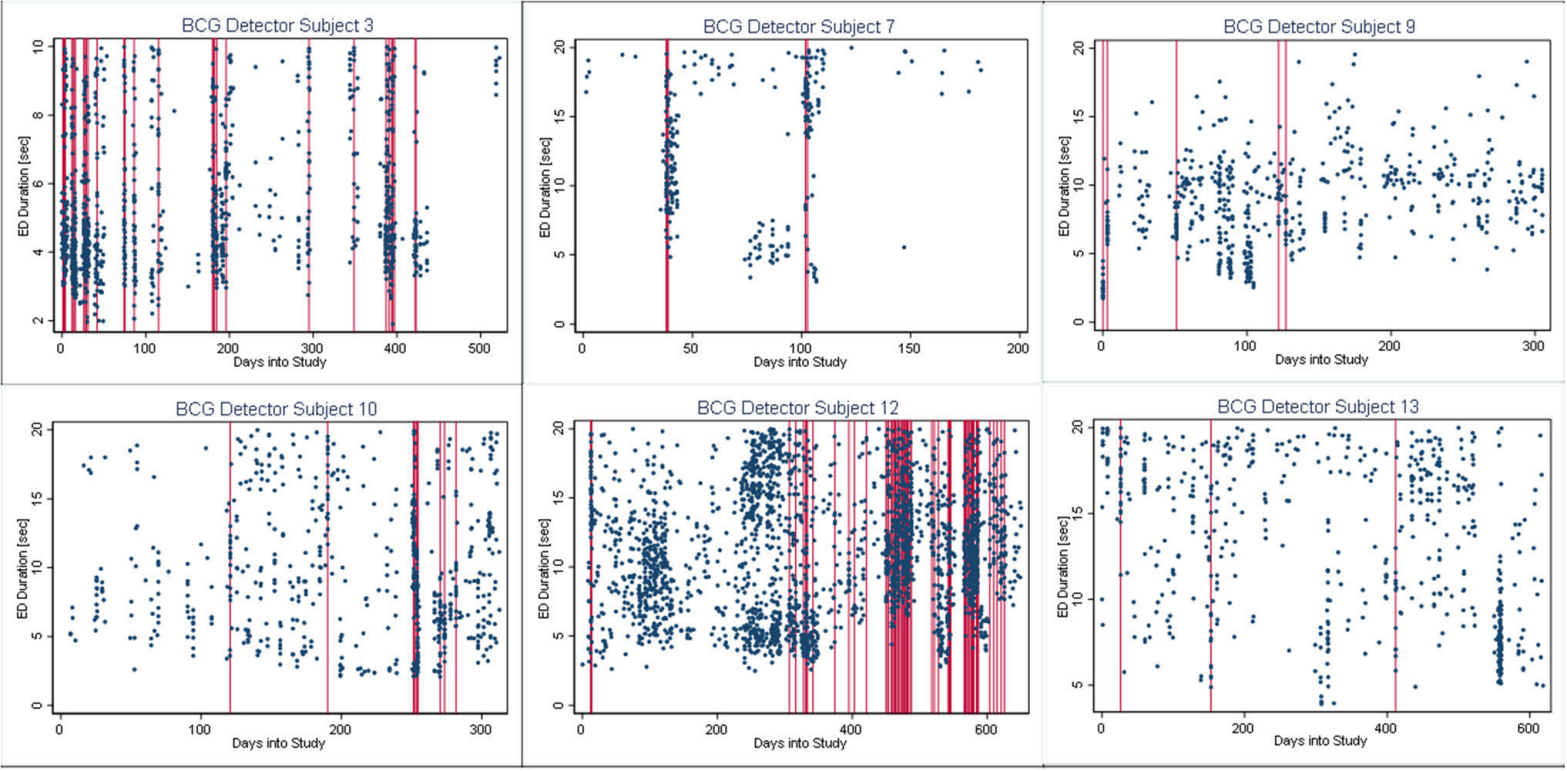
Burst-palisade plot of burst duration constrained grouping detector. Similar to Figure 4, the alignment of vertical lines and palisades indicates good agreement between the graphical method and the BCG detector.

**Figure 6:**
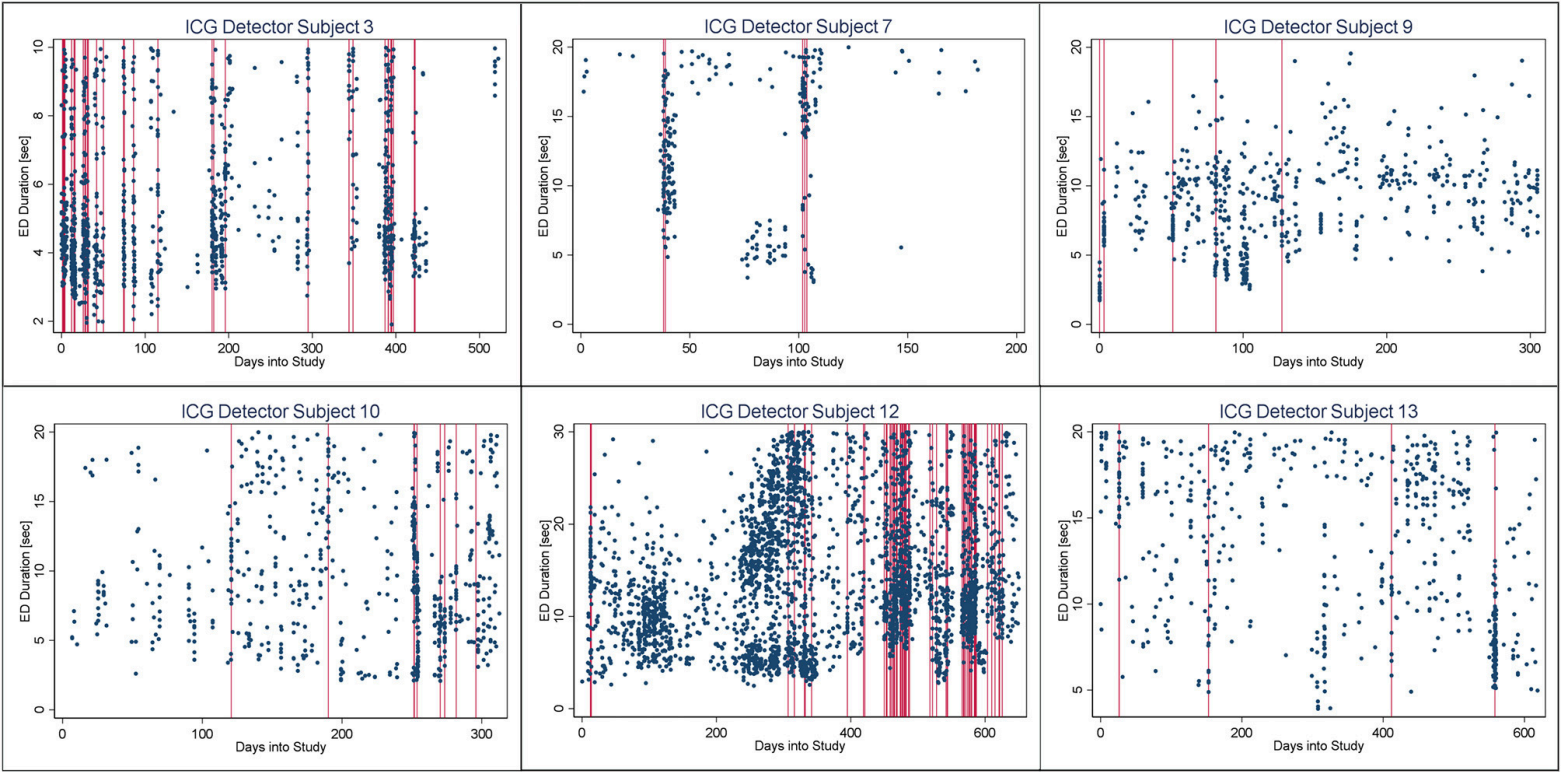
Burst-palisade plot of interseizure interval constrained grouping detector. Similar to figure 4, the alignment of vertical lines and palisades indicates good agreement between the graphical method and the ICG detector.

In Figures 4–6, we present plots showing the agreement between the modified scatter plot and three quantitative methods: the burst-palisade plots. Figure 4 is for the PCG burst detector, 5 for the BCG detector, and 6 for the ICG detector. The overlapping of Palisades of the modified scatter plot and vertical lines of the quantitative method indicates good agreement between the two methods in burst detection.

#### Concordance Correlation Coefficient

For all concordance analyses, the concordance correlation coefficient was ≥0.99 signifying considerably stronger than chance burst detector agreements with one another.

## Discussion

We have described graphical and quantitative methods to detect, characterize, and quantify seizure bursts. The graphical methods have revealed three patterns to identify bursts with visual analysis: the Palisade pattern, the Staircase pattern, and the Density band pattern. The quantitative methods have provided burst-detection algorithms to define and quantify bursts. We have also demonstrated agreement among different methods indicating the robustness of our approach. We tested our methodology in a group of 15 patients with drug-refractory focal epilepsy who underwent intracranial ambulatory EEG monitoring and detected six subjects with seizure bursts demonstrating the applicability of our methods (6). We present a comprehensive analysis of a single subject in this paper to highlight the details of our methodology so that it can be replicated and retested by researchers in the future.

Several authors have noted that seizures tend to cluster around certain time points, but there is no universal agreement on a definition of this phenomenon. The reported prevalence of seizure clustering ranges from 13 to 76% depending on the definition, the methodology of cluster detection (e.g., seizure diaries vs. video-EEG monitoring), and the cohort characteristics (1). The recognition and detection of seizure clusters have important implications for routine clinical practice. Seizure clustering has been reported to be a marker of antiepileptic drug resistance (4). Additionally, seizure clustering has been found to be associated with frequent hospital admissions, higher risk of postictal psychosis, and increased morbidity as well as mortality (1). Seizure clustering is perhaps an under-recognized phenomenon. Canine epilepsy is known to resemble human epilepsy and prolonged intracranial EEG recordings in dogs with focal epilepsy have demonstrated a tendency for most seizures to cluster (18).

Furthermore, clustering and bursting of seizures can potentially pose challenges in the presurgical evaluation due to inadequate sampling. When the subject has multiple seizure foci, as a result of clustering tendency, a series of seizures may originate from a single focus. If the video-EEG monitoring is not continued for a sufficient length of time, seizures from other foci may not be captured and inaccurate conclusions might be drawn regarding the localization as well as the suitability for epilepsy surgery. This pitfall is particularly relevant in drug-refractory epilepsy where clustering is a well-known association.

In keeping with theoretical concepts of spike bursts, we have introduced the term “seizure burst” to identify aggregation of seizures in the temporal domain. Conceptually and statistically bursts and clusters demonstrate some differences as well as similarities. Seizure bursts consist of both clinical as well as subclinical seizures, whereas seizure clusters include only clinical seizures. Conceptually, bursts are intrinsically connected with both time and rates. Bursts of events happen rapidly and in a repetitive style. Clusters can be repetitive too but the explicit context (e.g., time or space) is the unifying descriptor. Bursts usually occur within a shorter time window (minutes) as opposed to clusters measured over longer time spans (hours to days). Statistically, both clusters and bursts imply a memory of event timings so that interseizure intervals do not follow a Poisson distribution suggesting that individual seizures within a cluster or burst are not independent events.

It is important to compare the bursting phenomenon with the clinical concept of seizure clusters, which consist of a series of seizures, occurring in a group, with shorter than usual interictal periods. However, there is no universal agreement on the number of seizures and the time limit to which a cluster may be contained. The most widely used definition is ≥3 seizures within 24 h described by Haut (2). Other definitions include ≥3 seizures within 4 h in the epilepsy monitoring unit (19), an episode of multiple seizures distinguishable from the usual seizure pattern occurring within 24 h (in adults) or 12 h (in children) (20), and ≥3-fold increase in the perimenstrual period (catamenial seizure cluster) (21). From a statistical standpoint, previous studies have considered deviations from the Poisson distribution as evidence of clustering (2, 3). We have used a wider variety of methods, both qualitative and quantitative, to delineate and define seizure bursts. We also emphasize that seizure bursts described by us is primarily a mathematical delineation and the underpinning biologic mechanisms remain to be elucidated. Additionally, the clinical significance of seizure bursts warrants further research and we speculate, similar to seizure clusters, bursting may be a potential marker of drug-refractory epilepsy.

An interesting observation in Burst-palisade plots (Figures 4–6) is the evidence of occasional clustering of bursts, i.e., three or more bursts within close proximity of one another indicated by a group of vertical lines. Just as bursts are a congregation of seizures, bursts themselves seem to have the potential to exist in “groups” or “clusters.” It is interesting to note that the clusters of bursts themselves appear to be somewhat specific to the detectors, or the detectors have clustering specificity in regard to the detection of burst clusters. For example, around day 200 we see three bursts for PCG and ICG but four for the BCG detector. The lone burst after day 300 for both BCG and PCG is clearly two bursts detected by ICG (Figures 4–6). Given that bursts consist of both clinical and subclinical seizures, clustering of bursts is probably a reflection of clustering of clinical seizures (clinical seizure clusters). Further research is warranted to investigate the relationship between seizure bursts and clusters.

Using the three quantitative methods (PCG, BCG, and ICG), in the patient (ID 3), we found a mean burst duration (across all bursts) of 1,630 s, a mean ISI of 109 s, and a mean seizure duration of around 6 s. Thus, on average, we observe approximately 16 seizures per burst in our studies, and our lower cut-off for the number of consecutive seizures to comprise a burst was 10. These findings can be compared with the literature on the detection of neural spike bursts. For instance, Legendy posited that spike bursts could be identified as regions where the local or regional mean firing rate was significantly higher than the long-term mean rate (10). The change in rate, termed the Poisson Surprise, was deemed to arise when the long-term mean rate, applied (as the Poisson parameter) to a Poisson prediction model, failed to accurately predict a regional firing level, and the region was identified as a spike burst. Using the Poisson Surprise method, Legendy examined spike trains with a view to isolating bursting regions and found that the bursting spike rate, was 3–6 times higher (40 sec^−1^) than the average spike rate, contained 10–50 spikes per burst, and lasted for about a second. The Poisson Surprise burst prediction method bears some resemblance to our Precision Controlled Grouping (PCG) procedure discussed above.

Using a graphical method involving spike scatter plots where the ordinal spike number (y-axis) and time of spike occurrence (x-axis) are shown, Turnbull et al. noticed the appearance of vertical lines (“Strings”) apparently reflecting the “simultaneous occurrence” of groups of spikes (7). These vertical lines were identified as bursts of spikes. String plots of neuronal spikes are essentially the same as our Staircase plots (Figure 2) with the x and y-axes switched and we have shown how clearly these plots indicate the presence of seizure bursts.

The underlying mechanisms and dynamics of the bursting and clustering phenomena in the brain are not well-delineated. Several mechanisms have been postulated to explain the generation of seizure clusters. This includes inadequate postictal inhibition (22), inherent self-triggering capacity overcoming postictal inhibition (22, 23), and natural clustering due to scale-free dynamics of interseizure intervals (23). It is a matter of debate whether the seizures within a burst are causally related or they represent independent and distinct events. However, the bursting phenomenon is in line with the previous research demonstrating the long-range dependence of seizure timings (24). Further research to delineate the EEG features associated with seizure bursts and clusters would be valuable in clinical practice.

Perhaps the greatest strength of our investigation is that we have both identified and presented plausible accounts of novel features of seizure patterns in ambulatory patients with epilepsy. Ambulatory intracranial EEG monitoring can be considered the best tool to explore seizure bursts, as patients tend to miss seizure symptoms and seizure diaries are less reliable due to under-reporting (5). Allied to this, we have developed an array of qualitative (graphical) methods to highlight the presence of these features, and quantitative methods to automate their precise points of occurrence in the temporal domain.

We acknowledge some limitations of the study. Our methodology is described based on drug-resistant epilepsy and it may not be extrapolated to all epilepsy patients in general. However, we believe that drug-resistant epilepsy is probably the best model to study this phenomenon. Long-term ambulatory intracranial EEG monitoring is a research tool which is not available in clinical practice at present. Hence, further research is needed to test the applicability of our methodology to detect seizure bursts and clusters in routine clinical practice with common data sources such as seizure diaries and video-EEG monitoring. The emergence of long-term ambulatory EEG monitoring using subcutaneous electrodes is likely to provide better opportunities to apply our methodology in the future (25).

## Conclusions

Seizure bursts is an underrecognized and underreported phenomenon with potential clinical implications. Using long-term intracranial EEG data, we have described both qualitative and quantitative methods to detect and quantify the bursting phenomenon. We believe that our methodology will provide a framework for other investigators to advance their own studies of burst detection and we hope the tools we have described may simplify their investigations.

## Author Contributions

US literature search, data collection, data interpretation, drafting, and critical revision of manuscript. RB study concept and design, data analysis and interpretation, and critical revision of manuscript. MC study concept and design, data interpretation, and critical revision of manuscript. PK and DF data interpretation and critical revision of manuscript.

### Conflict of Interest Statement

The authors declare that the research was conducted in the absence of any commercial or financial relationships that could be construed as a potential conflict of interest.
