# Supplementary material for: Methods for the Detection of Seizure Bursts in Epilepsy
Source: Front Neurol. 2019 Feb 27;10:156. doi: 10.3389/fneur.2019.00156 (PMC6400839; doi:10.3389/fneur.2019.00156)
Supplement: Supplementary file 1 [file Data_Sheet_1.docx]

**APPENDIX 1**

**TECHNICAL DETAILS OF THE BURST DETECTION METHODS AND OPERATIONAL APPROACH TO THEIR USE**

This appendix describes the graphical and quantitative methods for burst detection in more detail than the manuscript presents. The hope is that enough information is presented to enable the interested reader to implement the schemes. To start, we remind that there are 3 graphical and 3 quantitative methods all with mnemonic titles to ease their recall and basis. We start with the account of the graphical methods and finish with the quantitative methods. At the conclusion of this appendix, we present operational aspects of the implementations.

***Graphical Methods***

**1. Modified Scatter Plot**

This display is heavily based on the string plots of Turnbull^7^ and is the preferred presentation format to offer an account of all seizures recorded during the entire ambulatory period of each subject-typically 1 or 2 years. To enable the concurrent display of seizure duration and interseizure interval the plot has one x-axis, time into the study [day], and two y-axes (hence the title Modified Scatter Plot), ln seizure duration (left y-axis), and ln ISI (right y-axis) both expressed in raw units of seconds.

The principle here was that if, compared to units of the x-axis (day), the seizure durations are short and the seizures are close to one another (i.e. the ISI is relatively short too), then we should see vertical structures in the display with the durations at the base of the vertical structure and the ISI’s in the upper section. We called these structures Palisades, and they are themselves distinct evidence of bursts. An important feature of the Modified Scatter Plots is that they lose little of the burst structure details along the entire monitored period.

**2. Staircase Plot**

We needed to complement the Modified Scatter Plot with another display enabling us to zoom in to burst observation segments to explore in greater detail regions potentially conflicted amongst the detection methods combined. This led us to the Staircase Plot. Here the x-axis is the ordinal position of seizures (first occurring to last occurring in the segment being explored) and the y-axis is the precise time of the seizure. Since bursts are structures where short duration seizures rapidly take place, they would be identified in our Staircase Plot as horizontal lines. That is lines which appear to consume very little time in our ambulatory account.

On the other hand, regions, where bursts cease occurring, would be characterized in terms of vertical lines, hence the title for this display style ‘Staircase Plots. Note that selecting alternate seizure regions is very conveniently negotiated here simply by choosing a different seizure order, or onset, range (e.g. seizure numbers 50 to 100, or seizure numbers 325 to 375 etc).

**3. Dropline Plot**

In section 2.2 of his 2005 paper, Turnbull points out that as the frequency of spikes increases their density, in a digital sense, also increases and we sought to explore whether Turnbull’s notion might also apply to seizure bursts. Here we plotted in the y-axis seizure duration and, in the x-axis, the ordinal seizure number. However, rather than simply plotting points, we plotted vertical lines from the seizure duration point down to the x-axis as droplines. Since the burst elements are short and in close proximity with each other, the increased density in the plots as a burst is clearly evident.

***Quantitative methods***

The 3 quantitative methods we developed to automatically detect seizure bursts each used a distinct approach. The goal of each was to produce a transformation of the data which would 1) render groups of seizures potentially undifferentiated within the groups, i.e. forming clusters of such seizures, and 2) rendering seizures from different clusters being well differentiated from one another. These clusters would form the basis of our bursts. The methods were referred to as Precision Constrained Grouping (PCG), Burst duration Constrained Grouping (BCG), and Interseizure interval Constrained Grouping (ICG).

**1. Precision Constrained Grouping**

Because the studies we were interested in were monitored for up to several years of duration, and because the events we were tracking (seizures within bursts) were barely seconds in duration (and separated by no more than a minute or so, ISI) it appeared that a plausible way of aggregating or clustering the seizures into (burst) groups might be by use of a precision controlled transformation of the seizure onset times.

Consider for example two seizures of duration say 10 seconds (from a typical burst) separated by about 70 seconds from each other and the burst occurring 16 months along the EEG sampling line from a subject. Clearly, this event (seizure) separation would be very difficult to detect. But, on the other hand, compiling such information of proximal seizures into groups would be a highly plausible challenge. Indeed, this is the principle behind our PCG detector. We now demonstrate this process step computationally using actual data from one of our subjects

This table shows a data segment (comprising 14 seizures) reflecting the actual operation of this procedure. Here ‘lstartt’ and ‘lnnew’ are respectively the single (32 bit) and double (64 bit) precision log transformation of the onset times of 14 seizures experienced by subject 3. Then, a statistical group command is used to help identify groups of seizures which are undifferentiated in conjunction with the singe precision manipulation. Finally, using assignment commands, it is possible to 1) enumerate the group sizes and 2) assemble the sequence of seizures within each group. This procedure was used successfully for each of the 6 subjects exposing seizure bursts.

*Critical elements of the code to produce bursts for PCG detector*

generate lstartt=ln(start) // Create grouping variable

generate double lnnew=ln(nnew) // Create high precision sorting variable

egen g=group(lstartt) // Create groups ‘g’

bys g: generate ng=_N // Create group size variable ‘ng’

sort g lnnew // Sort seizure within bursts

by g: gen sn=_n // Assign seizure numbers within bursts

list g ng sn lstartt lnnew in 1/20 // Display section of results

+--------------------------------------------+

| g ng ns lstartt lnnew |

+--------------------------------------------+

1. | 1 3 1 20.977775574 20.977774923 |

2. | 1 3 2 20.977775574 20.977775020 |

3. | 1 3 3 20.977775574 20.977775097 |

+--------------------------------------------+

4. | 2 2 1 20.977777481 20.977778181 |

5. | 2 2 2 20.977777481 20.977778251 |

+--------------------------------------------+

6. | 3 6 1 20.977779388 20.977779937 |

7. | 3 6 2 20.977779388 20.977779989 |

8. | 3 6 3 20.977779388 20.977780047 |

9. | 3 6 4 20.977779388 20.977780135 |

10. | 3 6 5 20.977779388 20.977780214 |

11. | 3 6 6 20.977779388 20.977780336 |

12. | 4 3 1 20.977781296 20.977780497 |

13. | 4 3 2 20.977781296 20.977780564 |

14. | 4 3 3 20.977781296 20.977780747 |

+--------------------------------------------+

**2. Burst duration Constrained Grouping**

This detector used a similar principle to that of the PCG detector. That is to find a transformation of seizure onset times which renders proximal seizure onsets (as in seizure bursts) undifferentiated from one another but differentiated from seizures which exist in other bursts. Here the transformation we used was a floored variant of integer division. The integer division expresses the resultant quotient as an integer. Our quotient comprised as the numerator the seizure onset time and as the denominator the maximum burst duration. For example, say we have two seizures at onset times 321342 and 321412 seconds (i.e. the seizure onsets are 70 seconds apart) and that our maximum burst duration (a detector constraint) is, say, 2400 seconds, then our floored quotient would be 133 for each of these seizures. Thus based on our approach these two seizures would be admitted into the same burst. The actual value we used for the maximum burst duration was 2400 seconds

**3. Interseizure interval Constrained Grouping**

This detector is similar to the one suggested by Legendy^10^, Martinson^11^, and Turnbull^7^. The basis of this detector is that a maximum interseizure interval (ISI) is set and that all sequences of seizures with ISI’s less than the set maximum are potentially accepted as comprising components of bursts.

One way to implement this procedure is as follows: first sort the data and retain only ISI’s less than the unacceptable value. The retained ISI’s are candidates to exist within bursts and our next step needs to assign preliminary burst status (a block indicator is created for this purpose) to the blocks of data without jumps (remember dropping the large ISI’s, and retaining only bursts with at least 10 seizures will lead to discontinuities in the initial data ordering).

We use sorting and grouping tasks next and this leads to the identification of an ordered collection of bursts with their size, and their encapsulated and enumerated seizures.

Our ISI constraint value was set to 600 (sec).

***Operational aspects of automated detection methods***

There are four features of seizure observations needing to be set for all burst detectors to function operationally: 1) the minimum number of seizures per burst, 2) log transformation of the seizure onset times, 3) the maximum time duration allowable for a burst, and 4) the maximum inter-seizure interval allowable between the seizures within bursts.

For the reasons offered in the “quantitative methods” section of our article, and for each of the burst detectors (PCG, BCG, and ICG), we set the minimum number of seizures per burst to 10. Additionally and exclusively for the PCG detector, we needed to log transform the seizure onset time. At this point, the PCG detector becomes operational and proceeds to a point of identifying bursts by simply finding groups of transformed seizure onset times with the same value.

The BCG detector additionally required specification of the maximum burst duration and our graphical information suggested that a value of 40 minutes (2400 seconds) would work. This being set (see also Karoly^6^) meant that operationally, bursts became detectable via a grouping of ‘floored’ division of seizure onset time by maximum burst duration.

Finally, for the ICG detector to become operational we required the maximum ISI (inter-seizure interval) to be set. Using raw averages of our graphically located burst ISI, an appropriate maximum ISI was estimated to be approximately 600 seconds. Then, sequences of seizures were counted as contributing to bursts until the ISI of either of the burst boundary seizures exceeded 600 seconds. All encapsulated ISI within bursts were less than 600 seconds.

The following table summarizes the stepwise approach to automated burst detection methodology.

| **Step** | **Do as follows** | **Time** | **Typical Departures^1*^** |
| --- | --- | --- | --- |
| 1 | Run code for each of the three Detectors: PCG, BCG, and ICG yielding all burst details for the subject. Each detector involves less than 20 lines of code^2*^ | Typically <1 sec for studies involving 5,000 seizures | <2%: total bursts detected  <1%: maximum seizures per burst |
| 2 | Run sentinel analyzer resulting in the assessment of the agreement between detectors | ~2 seconds per detector pair^3*^ | <1%: disagreement for the same number of bursts reconciled |
| **Step** | **Phase 2: Reconciling Quantitative results with Graphical Patterns (Bursts and Palisade Structure Alignment)** | **Results from respective Phase 2 step** | |
| 1 | If false positives are found (unequal number of bursts by detectors) explore Modified Scatter Plot to help locate missing burst(s)^1*^ | Isolation of temporal region where crowding or marginal palisades exists. | |
| 2 | Explore Burst-Palisade plots to confirm bursts detected with each detector are consistent with the Palisades | False positives and false negatives assignable to detectors | |
| 3 | Explore Staircase plots in regions (from sentinel analyzer) of contention | Marginal rises of steps, and/or narrow step shelves, may point to overcrowding of bursts beyond the resolution of specific detectors | |

1*: We have seen in our report that when the quantitative detectors’ reports agree in regard to the total number of bursts detected, the concordance of burst onsets (from the sentinel analyzer) is extremely high (>=0.99). However, there was indeed a small number of differences in total bursts detected (<2% per 65 bursts) when the detectors were not constrained to the same number of total bursts. This is not surprising when we reflect on the extremely diverse bases of operation of the detectors. There are two points to take away here: 1) there is likely to be small differences in burst reports by detectors developed from different motivations, and 2) by developing our exploratory tools diversely along both quantitative and qualitative lines we have assembled the most effective, flexible facilities to help us discern the true picture of bursts and their composition.

2*: We used Stata2015 to create the code for all aspects of this work. Stata is an in-core processor. This means that Stata is, to some degree, limited in regard to the dataset sizes it readily negotiates, but that its computing time, for modest dataset sizes, is well-suited to the task focus here.

3*: Each problem posed for the Sentinel Analyzer, in fact, involves sets of bursts for two detectors; a reference set (e.g. the bursts predicted by the PCG), and a scanning set (e.g. the bursts predicted by the BCG). Our assessment of the agreement between these scanned results was expressed in terms of the burst sentinel concordance values between the two detectors.
